# Supplementary material for: Effects of Compound Microecological Preparation Supplementation on Production Performance and Nutrient Apparent Digestibility in Hu Sheep from the Rumen Perspective
Source: Microorganisms. 2025 Apr 27;13(5):999. doi: 10.3390/microorganisms13050999 (PMC12114592; doi:10.3390/microorganisms13050999)
Supplement: Supplementary file 1 [file microorganisms-13-00999-s001.zip › microorganisms-3545922-supplementary.pdf]

Supplementary material

Supplementary Table S1. Statistical Analysis of Sample Sequencing Data Processing Results

Sample ID	Raw CCS	Clean CCS	Effective CCS	AvgLen(bp)	Effective(%)	Sample ID	Raw CCS	Clean CCS	Effective CCS	AvgLen(bp)	Effective(%)
A1	33923	33893	27926	1455	82.32	Con1	35489	35433	30313	1455	85.42
A2	36281	36248	31267	1455	86.18	Con2	37998	37967	33258	1454	87.53
A3	38354	38332	37547	1457	97.9	Con3	38556	38526	33073	1454	85.78
A4	33885	33856	29210	1455	86.2	Con4	40524	40496	39834	1456	98.3
A5	35426	35390	34902	1454	98.52	Con5	36839	36815	32762	1455	88.93
A6	39080	39053	38406	1449	98.28	Con6	34223	34213	33719	1458	98.53
A7	33102	33097	32974	1450	99.61	Con7	37032	37023	36803	1462	99.38
A8	35344	35330	34986	1464	98.99	Con8	33346	33313	32985	1448	98.92
A9	41381	41375	40686	1447	98.32	Con9	34880	34857	34404	1452	98.64
A10	35364	35353	35155	1447	99.41	Con10	39592	39555	33454	1454	84.5
A11	35225	35213	34961	1453	99.25	Con11	33903	33882	27131	1453	80.03
A12	33707	33679	33426	1460	99.17	Con12	32922	32884	27201	1455	82.62
A13	35210	35183	34987	1452	99.37	Con13	40268	40259	38158	144465	94.76
A14	34717	34677	34471	1453	99.29	Con14	37871	37830	35761	1454	94.43
A15	39985	39953	38629	1459	96.61	Con15	35372	35340	34479	1455	97.48
A16	32694	32667	28452	1456	87.03	Con16	35080	35027	34441	1454	98.18
A17	32768	32756	32360	1454	98.75	Con17	37468	37443	31791	1454	84.85
A18	38750	38736	38153	1455	98.46	Con18	38583	38512	37367	1452	96.85
A19	38714	38701	38213	1454	98.71	Con19	33683	33669	33362	1452	99.05
A20	40886	40877	40461	1473	98.96	Con20	39720	39697	39434	1467	99.28

Raw-CCS: Counts of identified CCS reads in the sample; Clean CCS: Counts of clean CCS reads (post primer removal and length filtration); Effective-CCS: Counts of effective CCS reads after chimeric reads removal; Average length(bp): Average length reads in the sample; Effective (%): Percentage of effective CCS reads in raw reads.

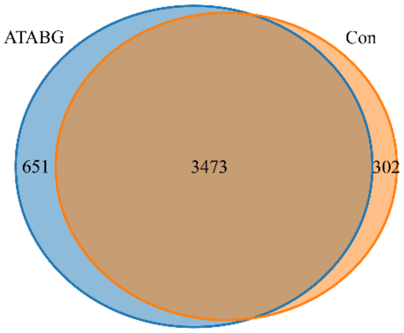

Supplementary Figure S1. Venn diagram of OTUs in bacteria of Hu sheep.

Supplementary Table S2. Composition of rumen bacteria at the phylum and genus levels %

Compositions	Groups[1]		*P*-value
	Con	ATABG	
phylum level			
Firmicutes	68.68±3.08[b]	77.15±2.35[a]	0.035
Bacteroidota	25.34±3.10[a]	14.38±2.90[b]	0.014
Proteobacteria	1.21±0.17[b]	2.63±0.48[a]	0.008
Cyanobacteria	0.73±0.21[b]	2.03±0.53[a]	0.028
Spirochaetota	1.52±0.29	0.86±0.24	0.087
Patescibacteria	0.67±0.14	0.39±0.06	0.073
Desulfobacterota	0.46±0.15	0.27±0.15	0.372
unclassified_Bacteria	0.17±0.04	0.50±0.17	0.068
Nanoarchaeota	0.03±0.02[b]	0.50±0.20[a]	0.022
Verrucomicrobiota	0.26±0.06	0.16±0.04	0.166
Others	0.91±0.14	0.84±0.15	0.725
Unassigned	0.01±0.01[b]	0.29±0.10[a]	0.011
genus level			
uncultured_rumen_bacterium	18.18±1.97	16.67±2.22	0.613
Ruminococcus	7.14±1.41[b]	14.84±2.62[a]	0.014
Rikenellaceae_RC9_gut_group	9.43±1.36	6.31±1.40	0.118
Christensenellaceae_R_7_group	7.26±1.17	7.12±2.05	0.951
Prevotella	6.53±0.96	4.21±1.03	0.106
UCG_004	2.55±0.56	4.33±1.02	0.136
unclassified_Ruminococcaceae	2.22±0.54	3.48±0.61	0.130
Quinella	3.74±2.08	1.80±0.92	0.400
NK4A214_group	2.68±0.38	2.44±0.57	0.730
Butyrivibrio	2.25±0.49	1.48±0.53	0.288
UCG_005	1.18±0.37	1.63±0.85	0.629
unclassified_RF39	0.55±0.10	1.01±0.20	0.052
Lachnospiraceae_NK3A20_group	1.99±0.50	0.95±0.32	0.086
Mogibacterium	1.40±0.22	0.97±0.22	0.176
Lachnospiraceae_AC2044_group	1.21±0.29	0.97±0.27	0.561
Pediococcus	0.86±0.22	1.06±0.54	0.733
Treponema	1.41±0.28	0.79±0.24	0.102
Family_XIII_AD3011_group	1.06±0.16	0.89±0.22	0.525
uncultured_Firmicutes_bacterium	0.49±0.09[b]	1.26±0.27[a]	0.010
unclassified_UCG_010	0.92±0.11	0.77±0.13	0.359

[1] The labels 'a' and 'b' in the same line indicate significant differences between groups (Wilcoxon rank-sum tests, $P<0.05$). The absence of labels indicates no significant difference.
